# Tempo and Mode in Evolution of Transcriptional Regulation

**DOI:** 10.1371/journal.pgen.1002432

**Published:** 2012-01-19

**Authors:** Kacy L. Gordon, Ilya Ruvinsky

**Affiliations:** 1 Department of Organismal Biology and Anatomy, University of Chicago, Chicago, Illinois, United States of America; 2 Department of Ecology and Evolution and Institute for Genomics and Systems Biology, University of Chicago, Chicago, Illinois, United States of America; Fred Hutchinson Cancer Research Center, United States of America

## Abstract

Perennial questions of evolutionary biology can be applied to gene regulatory systems using the abundance of experimental data addressing gene regulation in a comparative context. What is the tempo (frequency, rate) and mode (way, mechanism) of transcriptional regulatory evolution? Here we synthesize the results of 230 experiments performed on insects and nematodes in which regulatory DNA from one species was used to drive gene expression in another species. General principles of regulatory evolution emerge. Gene regulatory evolution is widespread and accumulates with genetic divergence in both insects and nematodes. Divergence in *cis* is more common than divergence in *trans*. Coevolution between *cis* and *trans* shows a particular increase over greater evolutionary timespans, especially in sex-specific gene regulation. Despite these generalities, the evolution of gene regulation is gene- and taxon-specific. The congruence of these conclusions with evidence from other types of experiments suggests that general principles are discoverable, and a unified view of the tempo and mode of regulatory evolution may be achievable.

## Introduction

Seven decades ago, the big tent of the Modern Evolutionary Synthesis was erected, and since then geneticists, paleontologists, ecologists, and their colleagues have been contributing from different angles to a more complete understanding of evolutionary processes. A cornerstone of the Modern Synthesis, Simpson's *Tempo and Mode in Evolution*, attempted to reconcile shorter timescale processes invoked by population genetics with the long-term patterns observed by paleontology, paying special attention to the tempo of evolution (the “when” and “how fast” of evolution) and the mode of evolution (the “how” and possibly “why” of evolution) [1]. The evolutionary questions we ask today drive ever deeper into these domains. How do changes in DNA manifest themselves as changes in an organism? Over what timescales do these processes unfold? Regulatory DNA—the sequences in the genome that control when and where protein-coding or RNA genes are expressed—may be a fruitful place to look for links between changes in DNA and novel phenotypes. At the very least, gene regulation is itself a type of phenotype, and is no less amenable than morphology to analyses that can detect evolutionary patterns and lead to the inference of evolutionary processes. Here, we apply questions of tempo and mode to gene regulatory evolution, inspired by Simpson's mechanistic insight that small changes are relevant to broad evolutionary processes, and that the effects of these changes will manifest themselves in different patterns over small versus large evolutionary timescales.

All evolution proceeds through descent with modification [2]. Transcription is regulated by sequence-specific binding of transcription factors and other proteins to enhancer and promoter DNA [3]. Therefore, transcriptional regulatory evolution consists of occasional (and occasionally functional) mutations in otherwise conserved transcription factors and *cis*-regulatory DNA elements. It is fraught with recruitment of existing regulatory interactions for novel functions and the turnover of sequences in the maintenance of existing functions [3]–[5].

Two unexpected discoveries propelled the study of regulatory evolution. First, the surprising degree of conservation of protein sequences between human and chimpanzee led to the hypothesis that the source of morphological disparity between the two species must reside in regulatory loci [6]. Second, studies of evolutionary developmental biology (“Evo-Devo”) discovered deeply conserved developmental regulators [5], including transcription factors like Hox genes [7] and master-regulator genes [8], [9]. Perhaps because of the striking conservation of transcription factors, conserved non-coding regulatory DNA became the focus of comparative studies of gene regulation [10].

However, the relationship between sequence conservation and functional conservation in regulatory elements is murky [11], [12], making studies of gene regulatory evolution difficult [13]. Functional changes have been attributed to single base-pair differences in otherwise highly conserved regulatory DNA [14], and functional conservation can remain where no sequence conservation is readily detected [15], [16]. Sequence comparisons alone can only identify certain types of regulatory conservation; functional assays are necessary to identify others [17]. Since regulatory functions are not readable via a well-established “code” [13], the prevalence and mode of regulatory evolution remain open questions that relate directly to those raised by the Modern Synthesis—what changes in the hereditary information? What effect on the organism?

A synthesis of a variety of experimental results could shed light on the prevalence of conservation versus divergence in transcriptional regulatory evolution, as it has illuminated other aspects of Evo-Devo [18], [19]. A number of studies using a range of techniques have been performed, including comparisons of endogenous gene expression [8], [20]–[24], ChIP-chip and ChIP-seq [25], [26] with binding profiles later compared between species [27], and gene expression studies in interspecific hybrids [22], [28]. Several exceptional studies independently determined gene networks underlying conserved traits in two different species [29]–[33]. Other studies focused on variations in post-transcriptional gene regulation via splicing [34], mRNA degradation [35], or microRNA-mediated silencing [36], which also regulate gene expression in important ways, and are probably subject to different constraints than transcriptional regulation. All of these studies contribute to a more complete understanding of how regulatory information is encoded in the genome and how it changes over evolutionary time. But with such disparate experiments to compare, it can be difficult to see the forest for the trees. A tremendous amount has been learned about the patterns and mechanisms of change in individual cases, but rather less can be said about general trends of regulatory evolution common across species.

There is one type of experiment that offers insight into regulatory divergence and has been performed for a number of genes in a number of species over the past several decades. It is methodologically simple and is feasible for non-hybridizing, non-model organisms. The experiment is an “enhancer swap” in which orthologous regulatory sequences from two species are each used to drive reporter gene expression in one of these species, so the expression patterns can be compared in the same *trans* background. This method yields easily interpretable results that may be compared between rather different case studies, so we looked at published experiments of this type. Ideally, finding global patterns could hint at evolutionary processes, and common conclusions across species and experimental paradigms could lead to general principles.

Nucleotide changes accumulate as two genomes diverge from a common ancestor. Enhancer swaps focus on changes within a single *cis*-regulatory element in one species, and changes to the loci that regulate it in *trans* in the other species. Some of these differences will be functional with respect to the expression of a reporter gene; others will be functionally mute. Enhancer swap experiments can distinguish between those two possibilities, because they ask only whether two different inputs (*cis*-regulatory sequences) give the same or different outputs (expression patterns of the reporter genes). We can therefore treat the molecular processes underlying transcription as a black box. In this way, enhancer swap experiments are conceptually similar to the experiments that deciphered the genetic code. The composition and function of the ribosome did not need to be understood in order to relate the sequence of synthetic mRNAs to the output of amino acid polymers [37]. Likewise, the output of gene expression from an enhancer swap can help us infer what information is encoded in a pair of divergent sequences, and whether their sequence differences have a functional effect on that encoding. Precise understanding of the molecular interactions controlling transcription is not necessary for these conclusions to be drawn.

By comparing swaps of a number of enhancers from a multitude of species, we can search for general patterns in the evolution of gene regulation. If patterns appear, despite case-to-case variation in experimentation as well as biology, they will suggest the existence of general organizational principles. We assembled a dataset comprised of 114 studies reporting 230 experiments (Table S1). The range of organisms that were compared with *Drosophila melanogaster* extends from its sister species to beetles, with whom flies shared a last common ancestor in the Carboniferous [38]. Swaps among nematodes were carried out between *Caenorhabditis elegans* and its closest known relatives as well as distantly related plant and animal parasites.

Only studies involving one of these two model systems were considered for two reasons. First, they anchor phylogenetic comparisons, which are necessary for insight into the *tempo* of regulatory evolution. Second, they offer the most precise spatial resolution of gene expression patterns, revealing the *mode* of regulatory evolution. Small-scale differences in expression might be common between [16], [39], [40] and within species [41]. Some minor differences in gene expression can have major consequences for organismal fitness [42]. Since the magnitude of pattern difference may therefore be a poor proxy for its fitness consequences, any and all differences in expression were noted. For the purposes of this review, we are agnostic about the fitness consequences and action of natural selection on these differences. Because these data are inherently variable, we present only the best-supported and most conservative generalizations.

**Figure 1:**
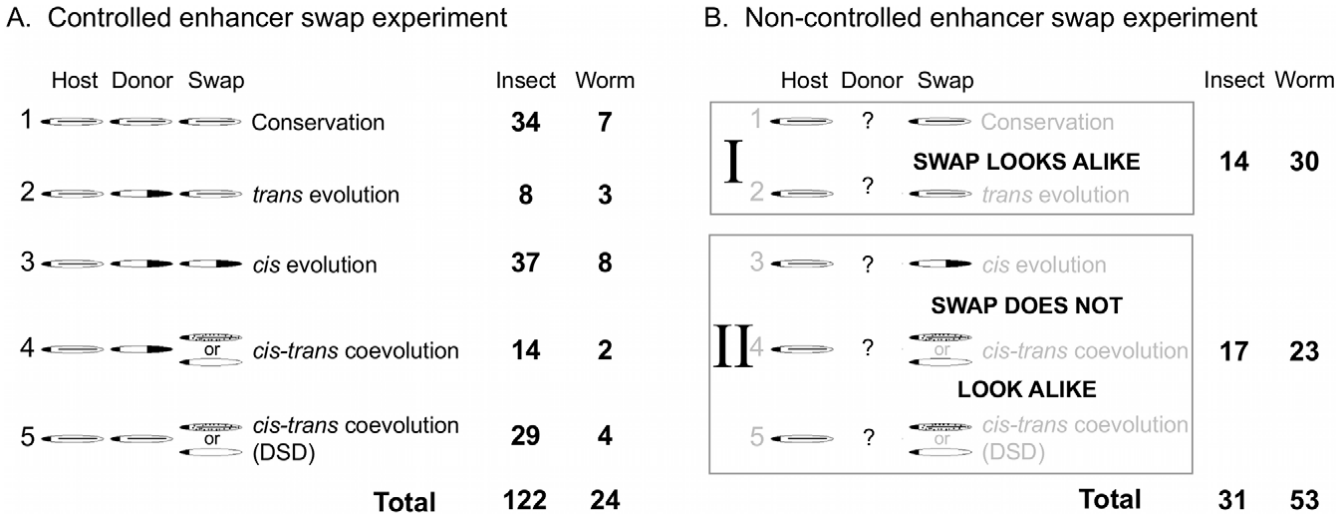
Categorized results of enhancer swap experiments. (A) Results of a controlled experiment can fall into one of five categories, depending on the endogenous expression patterns of the host, donor (from which *cis*-regulatory DNA was derived), and the swap (the donor DNA driving expression in the host organism). Cartoons depict schematic expression patterns showing similarity or difference, and are labeled with their biological interpretation. DSD, Developmental Systems Drift (Box 1). Numbers of swaps conducted among insects with *D. melanogaster* and among nematodes with *C. elegans* that fall into each category are shown. (B) Results of a non-controlled enhancer swap experiment can fall into one of only two categories, because information from the donor (or less often, the host) is missing. References and categorization given in Table S1.

For each study that reported an enhancer swap experiment, the species, gene, endogenous expression patterns (if described), the DNA regions used to drive expression, and the result of the enhancer swap were recorded (Table S1) and categorized according to a rubric of possible outcomes (Figure 1). While this sacrificed considerable richness of data reported in the original studies, it was necessary to compare only the elements that were common to all of the experiments. We also omitted those studies that used different approaches, although we have drawn from these other studies while interpreting the trends described here. As Simpson wrote in the introduction to *Tempo and Mode*, “The data will never be complete, and their useful, systematic acquisition is dependent upon the interpretation of the incomplete data already in hand” [1]. In this spirit, we hope that the currently available data can help to refine experimental paradigms of tomorrow.

## TEMPO: Evolution of Gene Regulation Is Rampant

First, we wanted to have an overall sense of the amount of evidence for regulatory evolution that has been observed. We therefore compared cases where conservation of the regulatory system is strongly supported (Figure 1A, Category 1) or suspected (Figure 1B, Category I) to cases of regulatory divergence, where some kind of evolution must have occurred (Figure 1A, Categories 2–5; Figure 1B, Category II). If divergence is observed only rarely, there would be little sense in looking for the mode by which it occurs. We could conclude that regulatory systems are indeed highly conserved in these animals.

However, we found that even with biases against the detection of evolution in gene regulatory systems (Box 2), over 60% (145/230) of enhancer swap experiments performed in both insects and worms showed divergence in *cis*, *trans*, or both. Comparatively less divergence (40/84; 48%) was observed in non-controlled experiments (Figure 1B) than in controlled experiments (105/146; 72%, Figure 1A). This can plausibly be explained by a priori assumptions about conservation. Some experiments were not controlled precisely because gene expression was known (or strongly suspected) to be conserved. In other cases, the conserved aspects of the pattern were more clearly described in the original study than nuanced differences, which could mask evidence of regulatory divergence. These nuanced differences are important, as in almost all cases regulatory divergence altered the expression pattern of a swapped enhancer without destroying it completely. For example, the enhancer of the *pes-1* gene of *C. elegans* and its ortholog in *Caenorhabditis briggsae* are expressed in apparently identical patterns in their species of origin (an unusually good control was performed), but when swapped between species, they drive weak expression in the same pattern, as well as ectopic expression in several additional cells [43]. Such ectopic expression would be easy to miss, and therefore might be more common than has been reported. The striking conservation touted by Evo-Devo studies is surely at work, but most regulatory mechanisms have been modified to a detectable degree.

**Box 1. Interpreting Experimental Results**

Individual experiments were coded according to the possible outcomes of an enhancer swap as shown in Figure 1 and Table S1. The most informative enhancer swap experiments are controlled. This requires the knowledge of endogenous expression patterns in both species—ideally of a given *cis*-regulatory DNA, but gene expression as determined by in situ hybridization can also be used (but see Box 2). Performance of the donor DNA in the host (the “swap”) can be compared to both endogenous expression patterns and categorized according to the type of evolutionary change it suggests. These categories explain our observations in the most parsimonious way; more complicated scenarios are possible, so we likely underestimate how much regulatory evolution has occurred.

Conservation is inferred when all three patterns are alike (Figure 1A, Category 1), although some changes in *cis* and *trans* will fail to affect expression in a way that is detected by these swaps, so evolutionary change will be underestimated for this reason as well. A host-like pattern is evidence for evolution in *trans*, since the *trans* factors of the host determine the pattern, and the *cis* elements of both species are functionally equivalent in the host (Figure 1A, Category 2). Conversely, a donor-like pattern is evidence for evolution in *cis*, since the donor *cis* element is sufficient, regardless of which species is the host (Figure 1A, Category 3).

If both *cis* and *trans* regulators of a given enhancer have diverged, malfunctioning combinations will be created in an enhancer swap. This happens when two (or more) regulators of a single enhancer change their regulatory roles and coevolve with each other, creating species-specific regulatory interactions. These interactions are incomplete in a swap since the host lacks coevolved *trans* factors that the donor DNA relies on for expression (Figure 1A, Categories 4 and 5). The swapped enhancer will fail to be expressed properly under these conditions. The result can either be failure of expression or ectopic expression. The differences can be dramatic or slight, but even slight differences are evidence of *cis-trans* coevolution [82]. Sometimes *cis-trans* coevolution leads to divergent expression patterns (Figure 1A, Category 4). Hypothetically, independent *cis* and *trans* changes could occur on each lineage. In other cases, the gene expression pattern is maintained despite evolution at the level of regulatory interactions in one or both lineages (Figure 1A, Category 5); this particular type of coevolution is known as Developmental Systems Drift [102].

Without controls, results of an enhancer swap are less informative (Figure 1B). If only one of the endogenous patterns (either donor or host) is known, the swap either does look like the pattern that is known (Figure 1B, Category I) or it does not (Figure 1B, Category II). While some complexities of the evolutionary dynamics are lost in such experiments, they can nonetheless distinguish most types of divergence from conservation. For this reason, they are still helpful to the goal of making a conservative estimate of how much evolutionary change has occurred.

**Box 2. Caveats and Biases**

The conclusions of this study must be viewed in the light of several caveats and biases that inherently complicate meta-analyses. In this box we discuss those that seem most pertinent to the conclusions we draw. Fortunately, most of them make our conclusions conservative, as they primarily compromise our ability to detect evidence of evolution.

*Choice of organisms*—Only swaps performed with *D. melanogaster* or *C. elegans* were analyzed, so that these species anchor the phylogenetic range of the studies and allow for evolutionarily meaningful, as well as precise, comparisons. Are these findings generalizable to other species?

*Choice of genes*—The enhancers used in these studies were chosen due to interest in the biology of the genes they regulate, so they may not be representative. About half of all genes tested in both *Drosophila* and non-*Drosophila* insects, as well as in *Caenorhabditis* nematodes, have regulatory functions, which may be greater than the proportion of regulatory genes in the genome.

*Multiply-tested genes*—For some genes, multiple enhancers were tested from the same species, or from multiple species. While no redundant experiments were counted (that is, the same enhancer from the same donor in the same host species), we did count all tests of different enhancers of the same gene. We removed the most-tested genes from the analysis (*eve* and *hb* in insects; *egl-17* in worms) to check if our conclusions stand, and they do.

*Choice of enhancer fragment*—Establishing homology between regulatory elements is difficult. In distantly related species, or in rapidly evolving enhancers, non-coding DNA is less likely to be alignable. The boundaries of regulatory elements may not be easily discernable, and sequence outside the limits of conservation may still have regulatory function [16]. Indeed, DNA fragments of different lengths can have different activities, even if they are centered on the same conserved region, so some differences that are attributed to species of origin might instead result from different sizes or boundaries of the tested elements, or from incorrect inferences of homology. When several enhancer lengths were tested, we counted only the results from the longest fragment.

*Power of detection*—Not every mode of evolution can readily be detected by an enhancer swap. For example, two *cis* changes in the same enhancer that compensate for one another will “travel” together through the experiment in donor DNA, hindering discovery [103]. While we believe that the potential experimental outcomes as shown in Figure 1 are comprehensive, not every experiment ends up in a published study. Particular interest in *cis*-regulatory evolution may bias the initial selection of genes; however, this would require remarkable intuition about the types of regulatory changes that lead to gene expression divergence. There may also be bias towards following up preliminary results that suggest evolution in *cis*, because the causal nucleotides will be found in a more restricted region than those acting in *trans*, for which the entire genome is potentially implicated. Additionally, when divergence causes the complete failure of expression of regulatory instructions from another species, the negative results may end up in a “file drawer” [104]. Negative results have been reported in only a few cases [59], [105], [106], making us suspect that the file drawer is not exactly empty. Many cases of regulatory divergence may be difficult to discern from experimental failure.

*Experimental precision and resolution*—Gene expression is a multidimensional phenotype, and not every dimension is measured with the same precision. In some cases in which it was quantified, gene expression levels differed while patterns stayed the same [42]. Apparently weaker enhancers that drove correct spatial expression were not counted as divergent unless they failed to rescue or their expression levels were quantified. Another aspect of gene regulation, timing of expression, was reported only in a minority of studies. If expression timing was noted to be incorrect in a swap, the enhancer was counted as divergent. Surely if more studies quantified expression and timing, more cases of regulatory divergence would have been discovered.

*Quality of control*—Some experiments are controlled not by reciprocal transgenics, but by in situ hybridization to determine the endogenous gene expression patterns. These controls are not ideal, since they reveal the total distribution of mRNA of a given gene, which may not be the same as the domain of expression driven by a single enhancer. In some cases, the expression pattern of a gene is known to result from the composite effect of multiple enhancers [54]. If the expression pattern of a given enhancer recapitulates part of the known expression pattern, it was coded as “alike” to that pattern. This surely missed some nuanced differences in enhancer specificity.

*Intentions of initial study*—Every study is done with unique motivations and its own particular questions, so we judged (subjectively) whether the language of the paper implied expectations that a given enhancer swap would show conservation or divergence (Table S1). These expectations were compared to the experimental outcomes coded as “conserved” (Figure 1A, Category 1; Figure 1B, Category I) or “diverged” (Figure 1A, Categories 2–5; Figure 1B, Category II). Experiments that expected conservation nonetheless observed divergence 50% (72/141) of the time. In some cases, conserved aspects of the expression pattern were emphasized in the text, but evidence of divergence was apparent in the data. Experiments expecting divergence observed it more frequently, in over 80% of the cases (73/89). These expectations were often based on known divergence in a gene regulatory cascade [59] or known divergence of a trait to which a particular gene had been linked [56], [107], [108]. We do not think the initial hypotheses are unduly influencing the outcome of experiments that predict regulatory evolution. The percentage of experiments that infer evolution remains relatively constant at about 60%. The percentage of experiments that expect evolution has increased over time to come into this range within the last five years or so (Figure S1). Happily, hypotheses are influenced by the evidence of previous experiments.

*“Man-bites-dog” bias*—Interest in gene regulatory evolution is not evenly spread among taxa or genes. In journalism, the saying goes that when a dog bites a man, that's not news, but when a man bites a dog, that's news! In terms of enhancer swap experiments, this may mean that genes whose expression is conserved among distantly related organisms, or divergent among closely related organisms, may be more scrutinized than cases of divergence over great evolutionary distances or conservation among sister taxa. If this is the case, it could inflate the apparent prevalence of divergence among close relatives and underestimate divergence among more distant relatives.

These results can be considered in the context of hybrid gene expression experiments that find a mixture of regulatory divergence and conservation genome-wide [35], [44]–[46]. Directed enhancer swap experiments targeting loci that are misregulated in hybrids could locate causal differences in expression. If these loci are misregulated when the enhancer from one species is swapped into the other, they are likely the sites of regulatory divergence. Alternatively, if misregulation is confined to the hybrid, then incompatibilities in the hybrid *trans* background are responsible. This may shed light on what proportion of hybrid defects is caused by divergence of gene expression in the two hybridizing species, and what proportion is caused by hybrid-specific regulatory defects. Another line of inquiry could be extended to the results of vertebrate enhancer swaps, where conservation of gene regulation has long been upheld [47], but recent interest has turned to cases of non-conservation in regulatory systems [48].

## TEMPO: Regulatory Changes Accumulate with Genetic Divergence

Given that regulatory evolution is common, we next examined whether the amount of divergence differed between nematodes and insects. We expected regulatory divergence to accumulate over time, but is this true across all the genes that were studied? Does divergence happen at the same or different rates in the two taxa? Does regulatory divergence happen so quickly as to lose phylogenetic signal within these timespans? Expectations for the answers to these questions are not straightforward. The overall proportion of conservation versus divergence that was observed among insects and nematodes by controlled enhancer swaps is similar, but when all experiments are considered, more divergence was discovered among insects (Figure 1). A more appropriate comparison should be made between groups of comparable genetic distance, like *C. elegans* and its closest relatives compared to *D. melanogaster* and the flies of the *obscura* group [49].

Evolution was inferred in about half of the enhancer swaps among nematodes sharing the last common ancestor of *C. elegans* and *C. briggsae* (34 cases of conservation, 30 cases of divergence). Among flies of the *obscura* group and *D. melanogaster*, nine cases of conservation and 12 cases of divergence were documented. The fraction of conserved to divergent cases does not appear to be different between these two groups (Fisher's exact test, *p* = 0.46).

It seems likely that evolutionary change in transcriptional regulation is correlated with overall genetic divergence. This hypothesis concurs with evidence from another line of experimentation. Microarray measurements of gene expression divergence among *Drosophila* species show a strong phylogenetic signal [50], [51]. More controlled evidence could be gathered by performing swaps of the enhancers of a set of conserved genes between *D. melanogaster* and *Drosophila pseudoobscura* and between *C. elegans* and *C. briggsae*. The analysis could also be extended to deeper branches. Repeating this analysis for other taxonomic groups could test whether the correlation between genetic distance and regulatory divergence holds across larger portions of the evolutionary tree. The analogous divergence in vertebrates is that between mouse and human [49], for which a number of non-controlled enhancer swaps have been performed.

## MODE: Divergence in *cis* Alone Is More Common than Divergence in *trans* Alone

Genes whose regulation was examined in controlled enhancer swaps either had conserved (Figure 1A, Categories 1 and 5) or differing (Categories 2–4) *endogenous* expression patterns. Endogenous expression pattern differences can be caused by regulatory divergence in *trans* alone (Category 2), *cis* alone (Category 3), or *cis-trans* coevolution (Category 4); enhancer swap experiments can tell the difference between these evolutionary modes, so we asked which was observed most frequently.

Enhancer swap experiments reveal considerably more *cis* alone than *trans* alone regulatory divergence (Figure 1A, numbers of experiments involving insects coded as Category 3 versus 2 are different as judged by a one-tailed z-test for proportions = 5.01, *p*<0.001), and a considerable amount of *cis-trans* coevolution underlying differing expression patterns (Category 4). Where in-depth data are available for individual genes, the excess of divergence in *cis* is also observed. The most-studied gene, *yellow*, showed divergence in *cis* alone in 15/26 cases [52]–[54], in *trans* alone in 3/26 [52], [55], and in *cis* and *trans* in 4/26 cases [55]–[57]; the remaining cases show conservation. This does not appear to be a gene bias against detecting *trans* divergence, because most genes for which *trans* divergence was documented also showed evidence of *cis* divergence [39], [58] or *cis-trans* coevolution [59] in other experiments. This suggests that a given gene is more likely to experience evolution in *cis* than in *trans*.

We do not think that bias explains the observation that more evidence for evolution in *cis* than in *trans* is observed (Box 2). A predominance of divergence in *cis* is consistent with the consensus of the earliest transgenic animal studies of regulatory evolution (reviewed by [60]). More recent and quantitative studies find a large effect of variation in *cis* to a given gene on its level of expression in yeast strains [61] and species [62] and animal strains [63] and species [64]. For example, an estimated 95% of human expression-QTLs are found in the 20-kb of sequence in *cis* to the transcription start site of a gene [65]. The conclusion that *cis*-regulatory evolution is observed more often than *trans*-regulatory evolution also agrees with theoretical arguments that *cis*-regulatory evolution should be common because it can break the pleiotropy of developmentally important genes [66], and these predictions are being born out by a growing number of studies linking *cis*-regulatory evolution to morphological change [18].

Since Categories 4 and 5 (in which coevolution between *cis* and *trans* has occurred) are well-represented, it is clear that changes in *trans* to the genes that were tested played an important role in the evolution of their regulation. Divergence in *trans* is common, but appears rarely to happen without coevolution in *cis*, as has been noted in hybrid studies [64], perhaps because evolutionary changes in *cis* quickly respond to *trans* changes. The dynamics of *cis-trans* coevolution are not well understood, and it is imperative that they be studied further, particularly with respect to how gene networks respond to single regulatory changes. A recent simulation and analysis of expression data suggested that *cis-trans* coevolution may play a larger role than has been previously recognized [67].

Coevolution between *cis* and *trans* may be more prevalent than is commonly thought, since methods that measure endogenous gene expression are not able to detect lineage-specific coevolution if it is constrained to preserve the same regulatory output [68], [69]. Enhancer swap experiments, especially with controls, reveal coevolution when the swapped enhancer expresses in a pattern that differs from either endogenous pattern (as in Figure 1A, Category 5). This is reminiscent of the phenotypes that result from transgressive segregation that is commonly observed in hybrids [62], [70]. When changes in a single lineage are balanced or compensatory, combining a subset of these changes with those that evolved independently in another lineage—whether by transgenics or by hybridization—can result in extreme phenotypes that do not recapitulate those of either parent. When these phenotypes occur in an enhancer swap experiment, they identify a particular pathway in which divergence has accumulated.

## TEMPO and MODE: Misregulation Increases with Phylogenetic Distance

**Figure 2:**
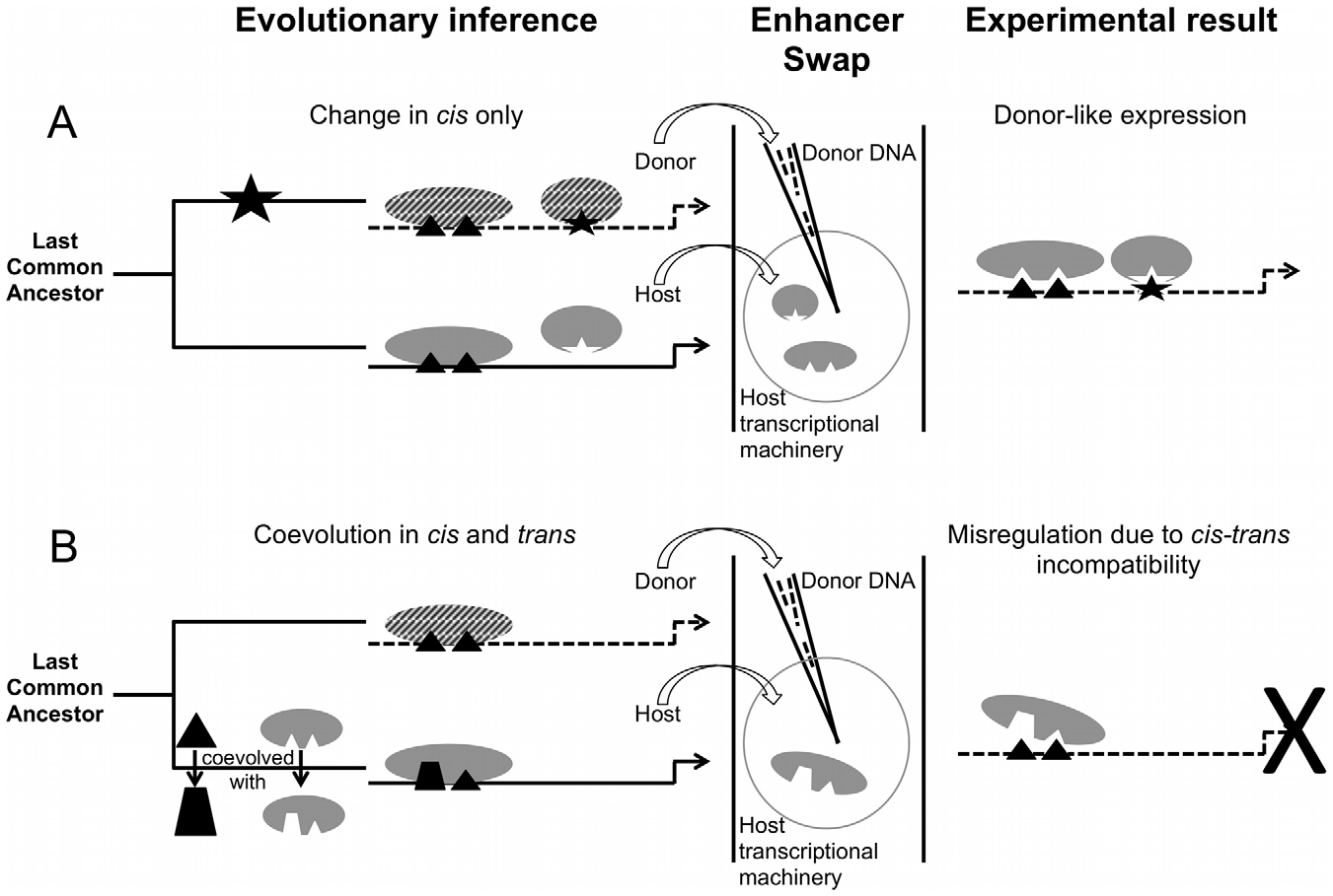
Divergence and misregulation proceed through different molecular mechanisms. (A) Evolution in *cis* alone, for example via binding site gain, can require only a single evolutionary step. Solid versus dashed lines/shapes represent different species. Binding sites (black) and transcription factors (gray) interact in the same way in both species, but a binding site (star) has been gained in the donor's *cis*-regulatory element, recruiting an existing regulator into the gene regulatory network. An enhancer swap combines this *cis* element (in needle) with the *trans* environment of the host (in circle), which is sufficient to drive a donor-like pattern. Divergence in *trans* alone is also possible, but is not depicted here. (B) *cis-trans* coevolution leads to misregulation upon swap. Binding sites and transcription factors interact in different ways in the two species. The donor *cis* element lacks the information that the host *trans* factors need to drive proper expression, so the pattern is not interpreted correctly. Not all *cis-trans* coevolution will take place through divergence of transcription factors and their binding sites as depicted; interactions with co-factors, the distribution of factors, etc. can also cause divergence in *trans* to a given enhancer (not shown).

Coevolution between *cis* and *trans* may constitute a distinct mode of evolution that unfolds with a slower tempo than *cis* or *trans* divergence alone. In Categories 2 and 3, an underlying regulatory logic is shared by both the donor and host. Evolution tweaked a parameter, a *cis*-regulatory element is lost for example [71], or a transcription factor is expressed in a new pattern [72], [73], but the components necessary to execute regulatory instructions work together in the same fashion in both species. The mechanism for interpreting instructions and giving an output has been preserved; the input changed, so the output changed (Figure 2A). Consequently, *cis*-regulatory instructions can faithfully be executed by *trans*-regulatory factors in a species-specific manner upon swap. Because variation of *cis*-regulatory elements or *trans*-regulatory factors exists even within species [74], [75], we hypothesize that divergence of the type shown in Categories 2 and 3 is more likely to appear between closely related organisms [76], particularly in genetic systems that underlie divergent traits.

In contrast, Categories 4 and 5 suggest a breakdown in the regulatory interactions controlling gene expression that we call “misregulation”, which results from lineage-specific *cis-trans* coevolution. The host *trans* environment cannot properly interpret the donor's *cis* instructions (Figure 2B). More evolutionary time is required to accumulate divergence in several members of a group of interacting molecules, as opposed to the single step that can be sufficient for divergence of the type shown in Categories 2 and 3. For instance, the boundaries of expression driven by the *eve* stripe 2 enhancers of *Drosophila yakuba*, *Drosophila erecta*, and *D. pseudoobscura* in a *D. melanogaster* host overlap precisely with native *D. melanogaster* EVE protein expression [77]; *eve* stripe enhancers from sepsid flies, however, drive expression in stripes that do not have coincident boundaries with *D. melanogaster eve* enhancers' stripe expression [15]. More functional divergence has accumulated in these enhancers between sepsids and *D. melanogaster* than has accumulated among *Drosophila* flies.

While it is possible that multi-gene regulatory networks can be polymorphic within species [78], it seems more likely that variable inputs into otherwise conserved networks are the seeds of regulatory divergence. If multiple interacting variants exist in a single population, the risk of maladaptive combinations arising in individuals could be substantial. Considerable gene expression variation exists within species, and finding the source of that variation will test our hypothesis: *cis* or *trans* variants alone affect most gene expression variation within and between closely related species, and *cis-trans* coevolution is more likely to accumulate as lineages diverge.

We predict that divergence of Categories 2 and 3 and misregulation of Categories 4 and 5 will evolve at different rates. Evolution in *cis* alone has been shown to be a distinct mode of regulatory change from evolution in *trans* alone, and they have been found to accumulate differently within and between species [63]. Perhaps examining the mode of *cis-trans* coevolution among more distantly related species will reveal additional information about how regulatory systems change over time.

To this end, we compared groups of greater and lesser genetic divergence. Misregulation is observed half as frequently as divergence within both *Caenorhabditis* worms and *Drosophila* flies (Table 1), which have a similar amount of genetic divergence (see above). The proportions are not statistically different between these two groups (Fisher's exact test *p*>0.9). Because enhancers from distantly related nematodes were primarily swapped in a non-controlled fashion, we do not have the resolution to distinguish Categories 2 and 3 from Categories 4 and 5 (Table 1 and Table S1). Our examination of divergence at greater phylogenetic distances therefore focused on the insects where better controls were performed. Enhancers from non-*Drosophila* insects expressed in *D. melanogaster* were misregulated over three times more often than they were diverged (Table 1); enhancers from other *Drosophila* flies expressed in *D. melanogaster* were misregulated just over half as often as they were diverged. One possible interpretation of this significant difference (Fisher's exact test *p*<0.001) between enhancer swaps among *Drosophila* flies and those involving other insects (Table 1) is that over time, regulatory systems that first change either in *cis* or in *trans* (Categories 2 and 3) accumulate additional changes that result in misregulation when swapped (Categories 4 and 5). This interpretation would be undermined if the types of genes used in swaps between distantly related insects were somehow more likely to be misregulated, rendering the two types of experiments incomparable. We do not believe this to be the case, as the experiments conducted at both phylogenetic distances were an almost perfectly even mix of developmental regulatory genes and structural genes (Box 2), with several genes tested at both distances (Table S1).

**Table 1:** Misregulation increases with phylogenetic distance.

Taxa of Comparison	Conservation (Category 1)	Divergence (Categories 2, 3)	Misregulation (Categories 4, 5)
*Caenorhabditis* worms	7	11	6
Non-*Caenorhabditis* worms	3 (Category I)	10 (Category 4, 5, II)	10 (Category 4, 5, II)
*Drosophila* flies	23	39	22
Non-*Drosophila* insects	11	6	21

If misregulation accumulates by the same processes that lead to Dobzhansky-Muller incompatibilities—divergence in multiple interacting molecules—there are theoretical [79] and empirical [80], [81] reasons to suggest they will increase dramatically over time. The timescales considered by the studies we included are much greater than those over which speciation occurs, but if the same principle of snowballing accumulation of divergence in interacting loci is at play, regulatory elements from more distant taxa are likely to show misregulation in a swap. We do not currently have enough information to test this hypothesis, but enhancer swaps targeted to a particular phylogeny and large enough gene set could do just that.

Coevolution between transcription factors and their targets has been implicated in misregulation [82], [83]. Whether the endogenous expression patterns are divergent (Category 4) or conserved (Category 5), the information contained in *cis*-regulatory sequences and the loci that regulate them in *trans* are coevolving. These results suggest that some well-studied cases of regulatory evolution underlying divergent traits between closely related organisms, like stickleback fins [71] and mouse coat color [84], may constitute a particular type of gene regulatory evolution. It proceeds quickly under directional selection by modifying preexisting regulatory logics. In other cases, the more time passes since divergence from a common ancestor, the more changes accumulate in the logic of a regulatory network [59]. This accumulation could also be caused by selection on the network in one or both species, or possibly as a byproduct of other evolutionary forces. The rate at which misregulation accrues in conserved traits versus divergent traits should be tested explicitly. If the distinction holds up, different modes of evolution may underlie divergent gene regulation and deeply conserved expression patterns.

## TEMPO and MODE: Sex-Specific Regulation Diverges Faster than Tissue-Specific Regulation

We next examined sex-specific genes, whose expression we expected to evolve fairly rapidly, as they were our best candidates for making the comparison between recently diverged and more deeply diverged taxa. Our hypothesis is that they will show evidence of divergence in *cis* or *trans* (Categories 2 and 3) among close relatives and misregulation (coevolution between *cis* and *trans*, Categories 4 and 5) at greater phylogenetic distances.

**Figure 3:**
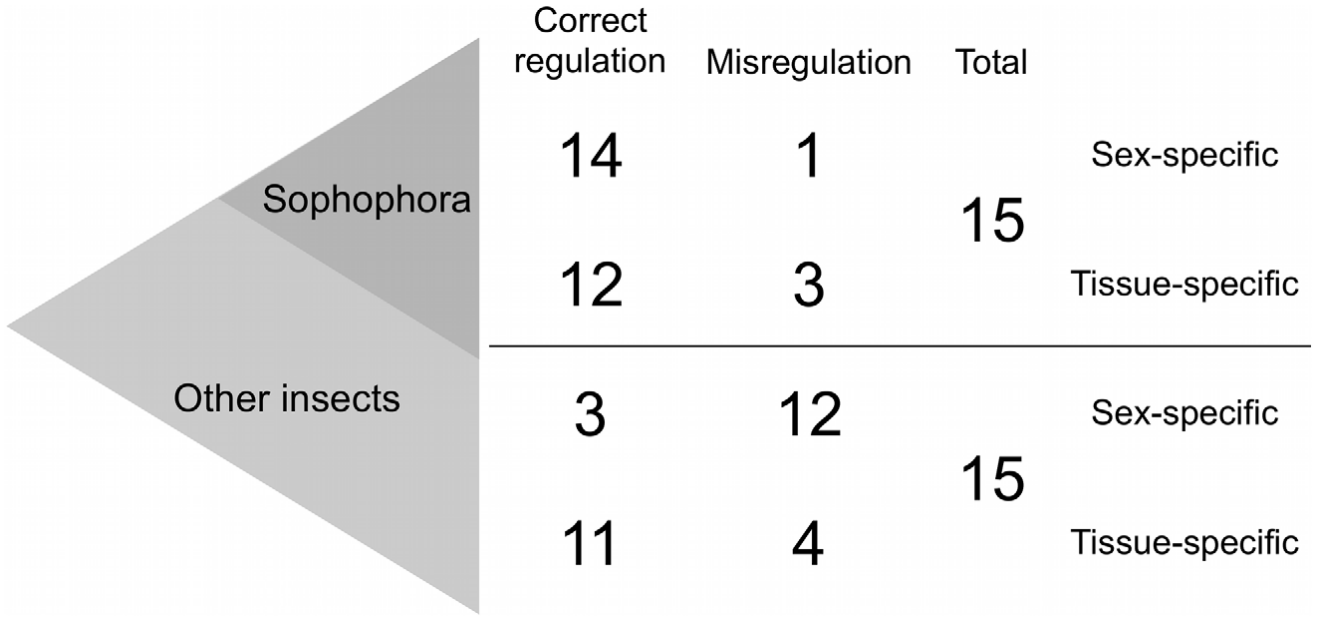
Sex-specific expression evolves faster than tissue-specific expression. When swapped into *D. melanogaster*, very few enhancers from other species of the *Sophophora* subgenus are misregulated with respect to sex or tissue. Enhancers from more distantly related insects, however, are misregulated more frequently with respect to sex than tissue when expressed in *D. melanogaster*. The total number of sex-specific enhancer swaps performed with *Sophophora* and with other insects is coincidentally 15 in each case.

Indeed, enhancers of genes with sex-specific expression are frequently misregulated when swapped into a distantly related organism (the trend described in the section above is robust to removing the sex-specific genes). Among flies of the same subgenus as *D. melanogaster* (the *Sophophora*, Figure 3), 14/15 enhancers drive proper sex-specific expression upon swap. In sharp contrast, enhancers from more distantly related insects only showed proper sex-specific expression in 3/15 cases. They were typically misregulated with respect to sex only, and not tissue (11/12 misregulated enhancers maintained tissue-specific expression).

Two conclusions can be drawn from this evidence. First, the tempo of sex-specific gene expression evolution is rapid. Of the 30 enhancers with sex-specific expression patterns, all but two [76], [85] show evidence of evolution in sex or tissue. This concords with what is known about the rapid evolution of sex-biased genes and their expression [45], [86]. The second conclusion is that when a gene is expressed in both a sex-specific and tissue-specific manner, the former evolves more rapidly. This observation reflects the modularity of gene regulation, as different functions are independently mutable, and apparently subject to different constraints. A similar phenomenon has been observed with the *cis* elements of distantly related nematodes in *C. elegans*, where stage-specific regulation was seen to diverge faster than tissue-specific regulation (Table S1, and reviewed by [87]). In even more distant swaps, heat shock promoters of *D. melanogaster* in *C. elegans* were found to retain inducibility upon heat shock, but not tissue-specificity [88]. Tissue, sex, stage, and inducibility are all aspects of gene expression that can apparently diverge at different rates.

The hypothesis that independent protein-DNA interactions determine different aspects of expression follows from these observations, and subsequent experiments have identified some of the regulatory information that may impart sex-specificity [85], [89]. Greater attention to sex- and stage-specificity of gene expression in other studies [90] will continue to reveal new insights into their evolution. Enhancer swaps of a set of orthologous *cis*-regulatory elements from progressively more distant species could test fidelity of sex-specific expression, and could point to particular transitions in sex-determination [91]. Nematodes, which have extensively modified reproductive modes [32], [49], [92], could be particularly appropriate organisms for such a study.

While the trend we observe is strong, there are some remarkable counterexamples. An enhancer from the closest relative to *D. melanogaster* that was tested by any study, *Drosophila sechellia*, was not expressed at all upon swap [85]. On the other hand, two chorion gene enhancers from the silk moth *Bombyx mori*, the most distantly related organism to *D. melanogaster* for which sex-specific genes were tested, were properly regulated with respect to sex, despite lacking orthologs in the *D. melanogaster* genome [93].

## MODE: Regulatory Changes Are Gene- and Species-Specific

The examples cited in the last section that counter the general trend we observe for sex-specific genes led us to ask whether lineage-specific contingency is rare or common. Will phylogenetic distance be able to predict whether a particular enhancer will function properly in a swap? Or will it be misleading to generalize the results of a single experiment to the behavior of regulatory elements of other genes or species?

Most evolutionary changes are highly specific with respect to the genes and species in which they occur. The dataset on which we are basing this conclusion is by its nature idiosyncratic, but the trend holds when we examine single genes or single taxon pairs. An instructive example is offered by the *yellow* gene, whose expression has been compared among multiple *Drosophila* species. Depending on the lineage, experiments found conservation, divergence in *cis* alone, *trans* alone, and coevolution between *cis* and *trans*, without much evidence to indicate shared changes [53]. This is true of other genes for which multiple enhancers from multiple species have been tested [15], [42], [82], [85], [94]–[97]. Conversely, when considering single species pairs (like *C. elegans* and *C. briggsae*, which were tested by most of the nematode experiments), studies of some enhancers found regulatory conservation and others found divergence in a gene-specific way (Table S1). Overall, most evolutionary changes observed by these studies occurred in taxon-specific ways, with ancestral states being modified independently on multiple descendant lineages [53], [54].

Both the abundance of regulatory evolution and the fact that it is widely observed on terminal branches accord with what is known about its mechanisms, further suggesting that species-specific divergence is not an experimental artifact of this hodge-podge assortment of enhancer swaps. Transcription-factor binding events are known to be species-specific [98], and may be subject to positive selection [99], [100] in their independent lineages. Terminal branches are also enriched for endogenous divergence in gene expression as measured by microarray [50], and endogenous divergence contributes to the divergence we observed (Figure 1A, Categories 2–4; and Figure 1B, Category II). Theoretical models of gene regulatory evolution suggest that many mutational paths can be followed by a given regulatory sequence while preserving its output [101]. The fact that general trends emerge from a big picture of gene regulatory evolution does not deny the importance of idiosyncratic changes in gene expression. These changes are important in that they are widespread. In some swaps, the enhancer from one species can be used to drive expression of the gene it normally regulates to test if it can rescue a host of the other species that lacks activity of the enhancer. One of the rare studies that did this type of experiment in flies found that the ability of enhancers from three species to rescue was inversely correlated to their phylogenetic distance from *D. melanogaster* [42]. While such an inverse correlation is unlikely to be found generally, this result is an excellent example of how regulatory changes accumulate on different lineages in a non-linear, unpredictable way. Such changes offer insight into the irregular nature of gene regulatory evolution. Far from accumulating equally in all loci over time, functional changes are episodic in a way that sometimes implies, and sometimes belies, phenotypic evolution. Functional tests of enhancer activity are therefore crucial.

## Conclusions

Whether or not they were looking for it, the majority of enhancer swap experiments performed over the past three decades on insects and nematodes have found evidence for gene regulatory evolution. This is true whether the output of the regulatory system is conserved or divergent. Divergent gene expression patterns are more often explained by evolution in *cis* than in *trans*; however, the abundance of *cis-trans* coevolution that is found by enhancer swap experiments means that both *cis* and *trans* evolution play an important role in shaping gene regulatory systems. Coevolution of this sort predominates among more distantly related organisms, especially with respect to sex-specific gene expression. In at least *Drosophila* and *Caenorhabditis*, regulatory divergence keeps pace with genetic divergence; however, counterexamples to this trend reinforce the lineage- and gene-specific nature of regulatory change. Functional tests of divergent regulatory sequences are necessary for understanding particular cases of regulatory evolution, as well as for bolstering the trends documented here. There is plenty of evidence of regulatory evolution written in animal genomes, and new techniques and directed investigations are bound to reveal more of it.

## Supporting Information

Figure S1 Changes in expectations and observations of evolution in published literature over time.
(PDF)

Table S1 Reference list and categories of experiments analyzed.
(XLSX)
