# Supplementary material for: Tempo and Mode in Evolution of Transcriptional Regulation
Source: PLoS Genet. 2012 Jan 19;8(1):e1002432. doi: 10.1371/journal.pgen.1002432 (PMC3261924; doi:10.1371/journal.pgen.1002432)
Supplement: Figure S1 — Changes in expectations and observations of evolution in published literature over time. (PDF) [file pgen.1002432.s001.pdf]

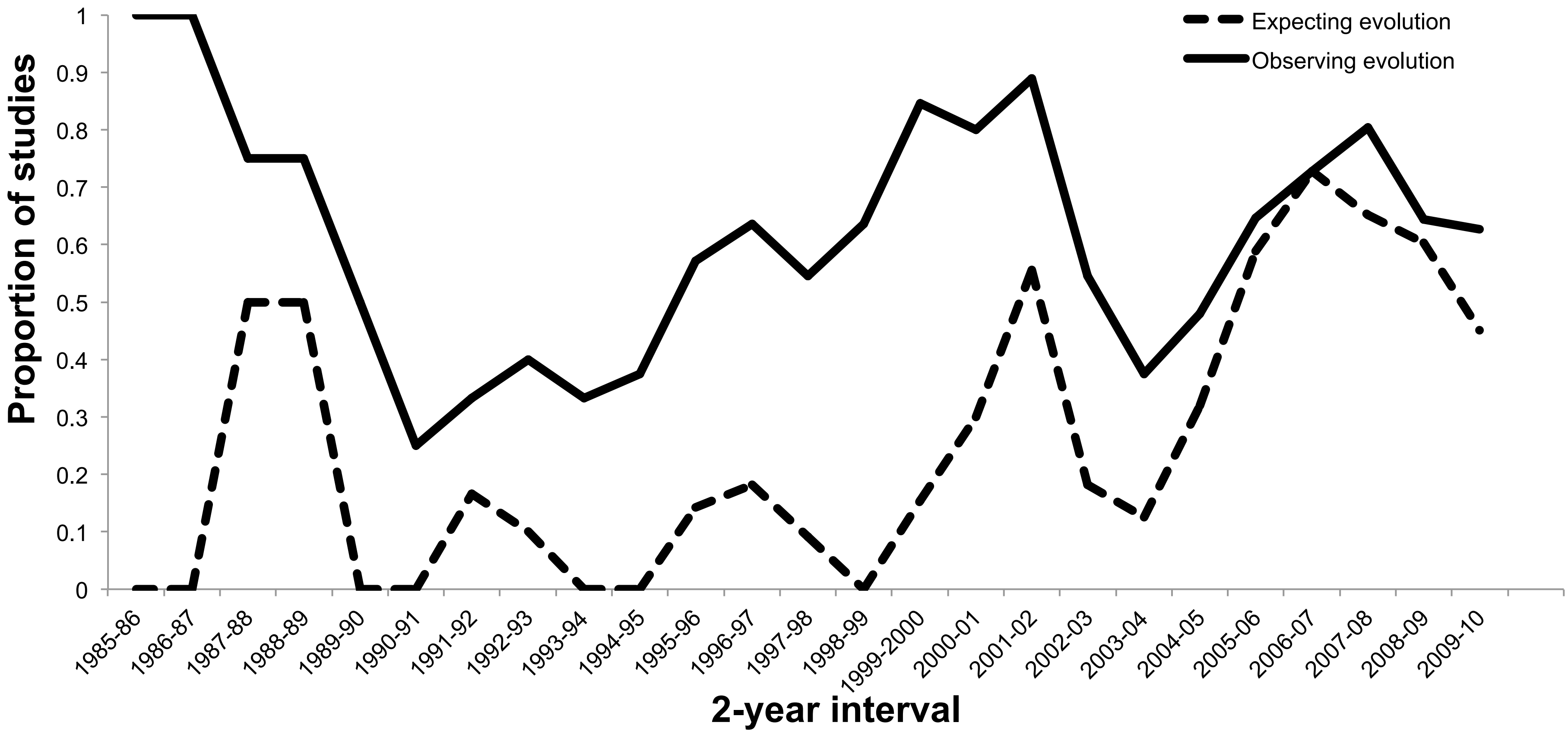

Figure S1. Changes in expectations and observations of evolution in published literature over time. Publications were sorted by year and coded for their expectations (dashed line) of evolution and observations (solid line) of evolution, as defined in Figure 1A, Categories 2-5 or Figure 1B, Category II. Proportions were calculated over sliding two-year intervals.
